# Textile sensors platform for the selective and simultaneous detection of chloride ion and pH in sweat

**DOI:** 10.1038/s41598-020-74337-w

**Published:** 2020-10-14

**Authors:** Luca Possanzini, Francesco Decataldo, Federica Mariani, Isacco Gualandi, Marta Tessarolo, Erika Scavetta, Beatrice Fraboni

**Affiliations:** 1 Department of Physics and Astronomy, University of Bologna, Viale Berti Pichat 6/2, 40127 Bologna, Italy; 2 Department of Industrial Chemistry, University of Bologna, Viale Risorgimento 4, 40136 Bologna, Italy

**Keywords:** Electrochemistry, Lab-on-a-chip, Sensors and biosensors, Nanoparticles, Polymers

## Abstract

The development of wearable sensors, in particular fully-textile ones, is one of the most interesting open challenges in bioelectronics. Several and significant steps forward have been taken in the last decade in order to achieve a compact, lightweight, cost-effective, and easy to wear platform for healthcare and sport activities real-time monitoring. We have developed a fully textile, multi-thread biosensing platform that can detect different bioanalytes simultaneously without interference, and, as an example, we propose it for testing chloride ions (Cl^−^) concentration and pH level. The textile sensors are simple threads, based on natural and synthetic fibers, coated with the conducting polymer poly(3,4-ethylenedioxythiophene):poly(styrene-sulfonate) (PEDOT:PSS) and properly functionalized with either a nano-composite material or a chemical sensitive dye to obtain Cl^−^ and pH selective sensing functionality, respectively. The single-thread sensors show excellent sensitivity, reproducibility, selectivity, long term stability and the ability to work with small volumes of solution. The performance of the developed textile devices is demonstrated both in buffer solution and in artificial human perspiration to perform on-demand and point-of-care epidermal fluids analysis. The possibility to easily knit or sew the thread sensors into fabrics opens up a new vision for a textile wearable multi-sensing platform achievable in the near future.

## Introduction

Wearable sensing technologies are attracting a growing academic and industrial interest thanks to the driving force of market demand and the prospective large impact on real life. Personalized healthcare and point-of-care medical assistance, together with fitness, represent the main fields of applications meeting the highest request for wearable biosensors.

The convergence of technical advancements in complementary research areas is making possible the development of wearable devices such as: (i) energy-harvesting devices^1–3^, (ii) energy-storage devices^3–5^ and (iii) sensors^6,7^.

On one hand, wearable physical sensors exhibit a high readiness level and include strain sensors to monitor physical movement^8^, motion^9,10^, heartbeat^11^ or respiratory rate evaluation^12^ and pressure sensors based on memory systems^13^, textile devices^14,15^ or skin-based architectures^16^. Moreover, bio-potential sensitive sensors can record skin potential that allows, for example, electrocardiograms^17–19^ and electromyography^20^.

On the other hand, wearable chemical sensors are at an early stage of development even if they represent a powerful tool to monitor human physiological parameters in a real-time, non-invasive and accurate manner. A number of new chemical sensors are proposed in the literature for the detection of biochemical markers like dopamine^21^, adrenaline^22,23^, cortisol^24^, glucose^25^, lactate^26^, phenolic compounds^27,28^, and electrolytes^29–32^ with the aim of providing new tools for monitoring human health, physical exertion, fatigue and mental accuracy. An interesting approach has been proposed by Gao et al. based on a fully integrated and mechanically flexible sensor array for multiplexed sweat analysis, which selectively and simultaneously measures perspiration electrolytes (sodium and potassium ions) and metabolites (glucose and lactate)^33^. A fully textile sensor device^34–39^ embodies the most advanced technological frontier to achieve complete flexibility, portability, non-invasiveness and lightweight towards continuous human body monitoring. Their wide spread use, in addition to a well-established manufacture background and their eco-friendly features, made textiles promising and cutting-edge materials for a new concept of smart sensors.

The possibility to integrate into a fabric an array of multiple textile sensors able to selectively detect different analytes would allow to implement a powerful textile multi-sensor platform, a sort of lab-on-fabric device.

The targets of textile chemical sensors are similar to the ones of wearable chemical sensors, i.e. the biomarkers in human perspiration, an epidermally available biofluid containing different compounds such as lactate, glucose, urea and electrolytes like sodium, chloride, calcium, and potassium ions^40^. Since they move from the bloodstream to the skin surface through sweat, they can be exploited to gain information about human physiological status and health^41^ with a non-invasive sampling performed outside the body. In addition, sweating can be stimulated on-demand with iontophoresis technique allowing for a continuous monitoring without external contamination and before analytes degradation.

Examples of chemical textile-based sensors were presented in the last decade to measure simultaneously various sweat parameters^42^, but only in the last 2 years preliminary multi-sensing systems based on functionalized textiles have been reported^6,43,44^. All reported systems are based on an electrochemical transduction that can be amperometric, potentiometric or mediated by an organic electrochemical transistor, and must be endowed with some additional elements such as reference, counter or gate electrodes. Therefore, several threads must be inserted in the fabric and must be immersed in the same electrolyte solution during sensing.

Our group has designed innovative sensors that are based on the semiconducting polymer PEDOT:PSS (poly(3,4-ethylenedioxythiophene):poly(styrene sulfonate)), which is a biocompatible, soft, flexible material able to work in aqueous environment. The sensing features of such devices are given by the functionalization with silver/silver chloride (Ag/AgCl) nanoparticles^45^ and bromothymol blue (BTB) dye^37^, for Cl^−^ concentration and pH detection, respectively.

Here we report on a further step forward in the development and optimization of such sensors as we realized two-terminal sensors fabricated onto single threads, based on PEDOT:PSS-Ag/AgCl and PEDOT:PSS-PEDOT:BTB, that can be easily woven or sewn into the same fabric, without requiring gate or reference electrodes for the analytes detection. The two-terminal thread sensors presented in this work, behave as an electrochemically gated device both for Cl^−^ and pH detection, merging the robustness of potentiometric-like transduction mechanism with a highly simple and feasible geometry without the need of a reference electrode.

Chloride and pH have been chosen as they are two common parameters analysed for wearable sensing applications owing to their relevance in human biomarkers monitoring^46^. As a matter of fact, their simultaneous detection allows for an overview of hydration status^47^, fatigue^48^, alkalosis^49^, metabolic reactions^50^ and the physiological conditions. It is worth to note that the Cl^−^ concentration in sweat (10–100 mM)^40^, which is a body fluid easier to access compared to serum (96–106 mM)^51^ or urine (15–40 mM)^52^, allows to make significant progress in developing textile electrochemical sensors without requiring sub-mM limit of detection. Moreover, typical pH values in human perspiration range from 4.7 to 6.6, but values up to pH 9 have been found in patients affected by cystic fibrosis^53^.

Three different kinds of thread, both natural (cotton and silk), or synthetic (polyester), were considered to evaluate possible effects of the textile substrate on the sensor performance.

We integrated such two-terminal sensors into an array of multiple textile sensors and we assessed their simultaneous and real-time selective sensing capability in artificial sweat. Moreover, they were sewn in a T-shirt and employed for the detection of Cl^−^ and pH in 1 mL of artificial perspiration. We further demonstrated the possibility to acquire raw data using a custom and portable read-out electronic from the novel multi-sensor textile platform here reported.

## Material and methods

### Reagents and materials

CLEVIOS PH 1000 suspension (PEDOT:PSS) was purchased by Haraeus (Hanau, Germany). (3-glycidyloxypropyl)trimethoxysilane (GOPS), potassium nitrate, silver nitrate, potassium hydroxide, acetic acid, boric acid, 85% phosphoric acid, phosphate buffered saline, sodium dihydrogen phosphate, L-histidine, sodium chloride, potassium chloride, 3,4-ethylenedioxythiophene (EDOT), bromothymol blue (BTB) were purchased from Sigma-Aldrich (St. Louis, USA). Ethylene glycol (EG) and polyethylene glycol were obtained from Carlo Erba (Cornaredo, Italy). All chemicals were of reagent grade. The Universal Buffer solution was prepared with 0.01 M H_3_BO_3_, 0.01 M H_3_PO_4_, and 0.01 M CH_3_COOH in 0.1 M KNO_3_. The artificial sweat formulation (ISO pH 5.5) was made up with 0.05% w/v L-histidine, 0.22% w/v NaH_2_PO_4_, and 0.5% w/v NaCl in distilled water.

### Apparatus

Scanning electron microscope (SEM, Cambrige Stereoscan 360) images have been acquired to investigate the microstructure, morphology, composition and coating features of the thread, both before and after the functionalization. The same instrument, coupled with an energy-dispersive spectrometer, was used to carry out the Energy-Dispersive X-ray Spectroscopy to determine the presence and distribution of the chemical elements in our samples. The accelerating voltage was 20 kV.

The electrochemical depositions were done using the Metrohm Autolab (Origgio, Italy) potentiostat in a three-electrodes cell, using the PEDOT:PSS-coated threads, a Pt wire and an aqueous saturated calomel electrode (SCE) as working, counter and reference electrodes, respectively. The electrical resistance of the threads was measured using a four-probe setup with the Keithley (Cleveland, USA) 2400 whereas, for the sensor characterization, the Keysight (Santa Rosa, USA) B2912A has been used to apply the required potentials. The accurate pH level has been measured using a commercial pH meter equipped with a pH glass electrode (XS Instruments pH 7). All the measurements were carried out in air and at room temperature.

### Fabrication of thread sensors

A conductive solution was prepared mixing CLEVIOS PH1000 suspension, ethylene glycol (secondary dopant), 3-glycidyloxypropyltrimethoxysilane (cross linker) and polyethylene glycol in the following volumetric ratio 77.1:13.8:0.9:8.2. After mixing by sonication CLEVIOS PH1000 suspension, ethylene glycol (secondary dopant) and 3-glycidyloxypropyltrimethoxysilna (cross linker) for 10 min, polyethylene glycol was added in the solution and kept under vigorous stirring for 20 min. The conductive ink was then warmed up in an oven at 70° aiming to increase the solution viscosity. Three different kinds of thread, that differ in the basic material composition, were employed as substrate: both natural (cotton: COT, and silk: SILK), and synthetic (polyester: POL). In addition, we introduced a second cotton yarn, named here COT2, to investigate the possible effects of a different fibers arrangement. It results in a tighter thread with a Metric Count of 2/30 instead of 2/50. The Metric cotton Count represents an indirect measure of the linear density and it is the number of threads hanks long 1 km to reach the mass of 1 kg. In order to deposit the polymeric mixture, the yarns were washed with neutral soap and distilled water and then immerged in the viscous solution and rolled around a stick with a constant velocity. The procedure was repeated until the thread surface was evenly covered. The modified thread electrodes were left to completely dry in an oven before proceeding with the next fabrication steps. The total amount of conductive polymer deposited on 2 cm long thread is about (0.5 ± 0.2) mg and it might slightly vary for different fibers. A proper functionalization process has the purpose to make the conductive textile thread highly sensitive and selective towards the target biocompound.

The chloride ion thread sensor was realized by electrochemically depositing Ag/AgCl nanoparticles using a three-electrode cell unit. Two consecutive steps compose the procedure: the Ag NPs were deposited dipping the 2 cm long conductive thread, in 0.1 M AgNO_3_ solution and applying a potential of − 0.2 V for 60 s. The device was rinsed in distilled water, dried and then immersed in 1 M KCl where the Ag/AgCl composite particle have been formed by applying a bias of + 0.6 V for 60 s. According to our previous work^45^, the Ag/AgCl NPs are composed by a core of silver, deposited during the first step, and an external shell of silver chloride, created throughout the second deposition step.

The polymerization solutions (PS) used to deposit PEDOT:BTB on conductive threads was prepared mixing 10 mM EDOT, 1 mM PBS with pH 7.0 and, 1 mM of BTB in 0.1 M KNO_3_ aqueous solution following a previously reported method^37^. The solution was stirred for 20 min. The thread was immersed in 25 ml of the described solution and cyclic voltammetry was carried out, sweeping the potential from 0 to 1 V applied vs SCE with a scan rate of 0.1 V/s for 10 cycles. The samples/threads were finally rinsed in DI water.

## Results and discussion

### Electrical and morphological characterization

**Figure 1:**
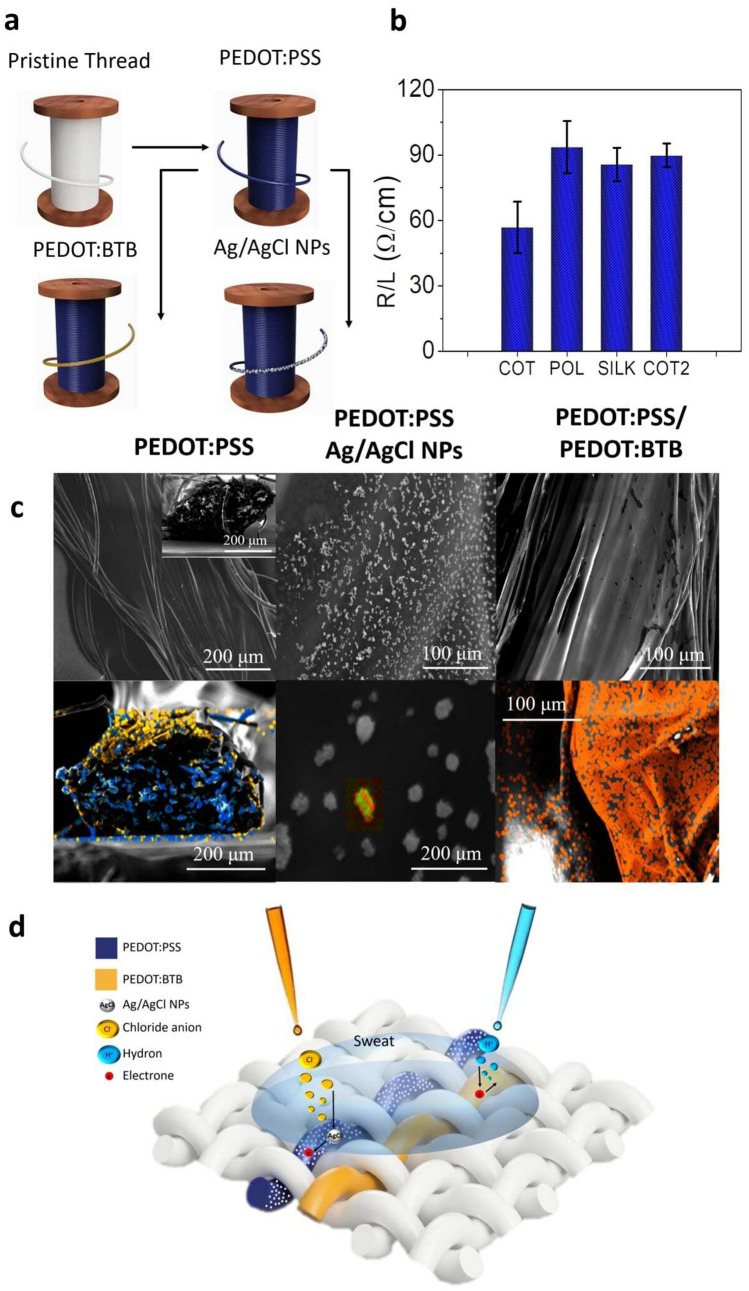
Fabrication and characterization procedures: **(a)** Schematic representation of the conductive thread before (top) and after (bottom) the electrochemical deposition processes; (**b)** average resistance value of the four different PEDOT:PSS-coated yarns measured with a four probes set-up; (**c)** SEM–EDX images of cotton thread (COT2) covered with PEDOT:PSS (left: yellow = sulphur group; blue = carbon), Ag/AgCl NPs (middle: red = chloride; green = silver) and PEDOT:BTB (right: orange = bromide). The images on bottom show the corresponding EDX analysis. (**d)** Schematic representation of the working principle of the thread sensors able to simultaneously detect, without interferences, Cl^−^ and pH level in sweat.

Figure 1a shows a schematic drawing of fabrication and functionalization steps of the sensitive threads for chloride anions and pH. Briefly, the commercial threads were covered with a PEDOT:PSS layer by physical deposition, in order to fabricate yarns that exhibit a semiconductor behaviour. Then, the transducing materials (Ag/AgCl for Cl^−^ and PEDOT:BTB for pH) were electrochemically deposited on the conductive polymer. The threads are purchased in local market and can be sewed into a fabric with a standard sewing machine or an industrial textile system thanks to their completely textile nature. This easy and low-cost fabrication process offers both high handling feature and conductive behaviour. After the PEDOT:PSS coating, the electrical resistances of the threads for the different fibers were measured using a four-point probe set-up, placing the probes one centimetre apart from each other. Four different types of threads have been tested: COT (pure cotton), POL (polyester) and SILK (pure silk) threads show comparable values (Fig. 1b) of (56 ± 9) Ω/cm, (90 ± 10) Ω/cm and, (85 ± 7) Ω/cm, respectively, while COT2 presents a resistance value of (89 ± 5) Ω/cm (see paragraph above for more details about fiber composition). Two bundles twisted together compose all the threads. Two different cotton threads were studied to investigate the possible effects due to the different numbers of fibers that compose the threads. We hypothesize that more PEDOT:PSS is present on COT thread since it fills also the spaces among the fibres on the surface (see the next paragraph for more details), causing a lower resistance. In order to check the success of the fabrication procedure previously described, Scanning Electron Microscopy was employed to obtain images of the four yarns coated with the polymeric mixture and after the electrochemical deposition of Ag/AgCl NPs and PEDOT:BTB. One cotton fiber is reported in Fig. 1c while the others in the Supplementary Fig. S1. No substantial difference was found among the four coated yarns, where the semiconducting polymer PEDOT:PSS mainly covers the outside of the thread with an average thickness of (12 ± 2) µm, as highlighted from the cross-sections in the inset of the left image. This is confirmed by Energy-Dispersive X-ray Spectroscopy (EDX) on cotton (bottom left image in Fig. 1c), which shows the presence of sulphur only in the outer shell of the thread (yellow), while carbon is present everywhere in the sample. In the pictures of PEDOT:PSS modified with Ag/AgCl, NPs are uniformly deposited on the surface of the polymer-coated threads having various size ranging from 300 nm to 30 µm. EDX confirmed the results of our previous work^45^, highlighting that the inner part of the nanoparticles is composed by silver (in green), while the outer shell is made of AgCl (chloride is represented in red in the image), thus being present in lower amount. The PEDOT:BTB electrodeposition resulted similar for all the fibers, only slightly changing their surface that looks rougher than before electrodeposition. The EDX map shows that bromine is uniform on the surface (in orange), suggesting that PEDOT:PSS is covered by a continuous layer of PEDOT:BTB. The SEM/EDX investigation demonstrates that modification was accomplished on all substrates.

### Working principles

The single yarn sensors work thanks to the potentials that are generated by spontaneous and reversible electrochemical reactions involving the sensing materials and the analytes. It is well known that an Ag wire covered by AgCl exhibits a reproducible and well-defined electrochemical potential (E) with respect to a reference electrode, which depends on the concentration of chloride ions in the solution. The Nernst equation describes this behaviour:

$$E={E}^{o}- \frac{RT}{nF}\,\mathit{ln}\,{a}_{{Cl}^{-}}$$

where E^o^, R, T, n and F are the standard potential of the redox couple, the gas constant, the temperature, the number of exchanged electrons and the Faraday constant, respectively. ${a}_{{Cl}^{-}}$ is the activity of chloride ion, but it can be replaced for practical purposes by [Cl^−^] as long as the experimental set-up ensures a constant activity coefficient. Since the Ag/AgCl NPs are in intimate contact with the semiconducting polymer, they play as nano-gate elements and the generated potential modulates the conductivity of the PEDOT:PSS. When the Cl^−^ concentration increases or decreases, the Ag/AgCl NPs potential must change in agreement with Nernst equation. Since the NPs are in electrical contact with PEDOT:PSS, a current will flow between the two materials until they reach the same potential value. This phenomenon changes the charge carrier concentration in the conductive polymer and thus its conductivity. The Fig. 1d shows a schematic representation of the working principle of the sensing threads. This process can be addressed as an electrochemical gating that was thoroughly described in our previous work ^45^. When BTB is used as the counterion, the acid–base equilibria involving its protonation and deprotonation impact on the stabilisation of the conducting form of PEDOT^37^. Overall, a redox equilibrium is established between the PEDOT^+^/PEDOT couple that depends on the solution pH, thanks to the pH dye doping agent:

$$2{vPEDOT}^{+}:{BTB}^{2-}+{e}^{-}+{H}^{+}\leftrightarrow PEDOT+ {PEDOT}^{+}:{BTB}^{-}$$

Also in this case the potential is described by Nerst equation:

$$E={E}^{0}+0.05916\, \mathrm{log}\,\frac{\left[2{PEDOT}^{+}:{BTB}^{2-}\right][{H}^{+}]}{\left[PEDOT\right][{PEDOT}^{+}:{BTB}^{-}]}$$

where [2PEDOT^+^:BTB^2^], [PEDOT] and [PEDOT^+^ + BTB^−^] are the concentration of 2PEDOT^+^:BTB^2^, PEDOT and PEDOT^+^ + BTB^−^ inside the polymer, respectively.

The transduction mechanism, originating the two terminal sensor response was discussed in a recent work^54^. Briefly, the electrochemical potential that is spontaneously generated at the PEDOT:BTB/electrolyte interface can be used to modulate the conductivity of the underlying PEDOT:PSS layer, in analogy with the electrochemical gating described for the Ag/AgCl NPs. The two terminal sensors enable to perform a reliable, sensitive and quantitative measurement, with the great advantage of using only two terminals connected to a single yarn to extract a significant measure of pH or chloride concentration.

### Single and multi-thread detection

**Figure 2:**
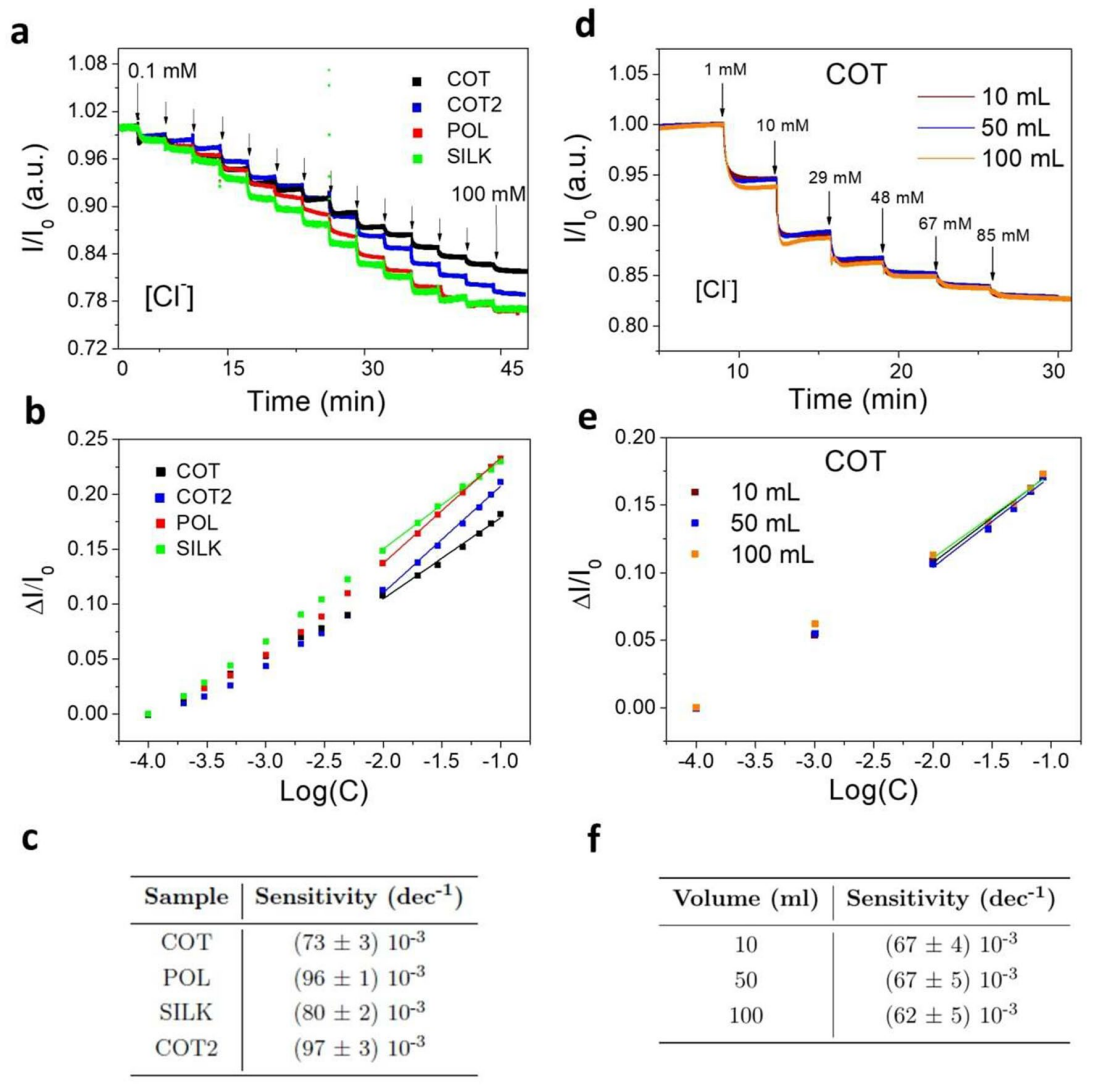
Single-thread chloride sensors characterization and volume effect: current signal acquired with a source meter for the different yarns immersed in 10 mL of 0.1 M KNO3: **(a)** I/I0(t) and **(b)** normalized current (I0 − I)/I0 vs the logarithm of Cl^−^ concentration from 0.1 mM to 100 mM of chloride concentration range. The potential applied to the two-terminal textile device is 0.1 V. The linear fit is performed in the physiological relevant concentration range for human sweat, that is between 10 and 100 mM. The sensitivities reported in table **(c)** are calculated in the same operational range. Example of the effect of the volume in the sensor response for COT: **(d)** I/I0(t) and **(e)** normalized current (I0 − I)/I0 vs the logarithm of Cl^−^ concentration from 1 to 115 mM of chloride concentration range. Three different solution volumes are presented: 10 mL, 50 mL and 100 mL. The linear fit is performed in the physiologically relevant concentration range for human sweat, that is between 10 and 115 mM. The sensitivities at different volumes of a COT yarn sensor are shown in table **(f)**.

As a first example of the sensor calibration, a long-term measure was carried out dipping the threads in 10 mL of 0.1 M KNO_3_ for the different yarn types (Fig. 2a). We changed the chloride anions concentration by adding selected volumes of 2 M KCl under a soft stirring. Each different fiber was tested in a Cl^−^ concentration range from 0.1 to 120 mM, in order to match the Cl^−^ content in human sweat, which is 10—120 mM^40,41^. In all cases, a linear fit between the normalized current variation (ΔI/I_0_) and the logarithm of [Cl^−^] was obtained in the physiological relevant range. All the calibration plots are linear (R^2^ > 0.97) in agreement with a potentiometric-like transduction, which is described by Nernst equation, as reported in the previous section. Figure 2b shows the data collected, with the value of the current at 0.1 mM [Cl^−^] used as I_0_. The points are reported without vertical error bars since the relative error is less than 1%. The table in Fig. 2c shows the extracted sensitivity values in which the error is the one associated to the slope derived from the linear fit. To prove the ability of our sensors to have reliable and repeatable sensitivities in different volumes, we tested the threads in different volume amounts of 0.1 M KNO_3_. As an example, Fig. 2d shows performance of COT, reporting a perfect superposition of the normalized currents for increasing volumes of the solution. This fact is also highlighted by the complete agreement among the sensitivities, as reported in Fig. 2e and in the table of Fig. 2f, the robustness and the high repeatability of the measure. In order to quantitatively assess and compare the performance of single thread sensors in the detection of Cl^−^ concentration and pH, we have separately evaluated them in 10 mL of Universal Buffer (UB), which allows stabilizing the pH values and to obtain well-controlled variations due to the additions of an acid or base.

**Figure 3:**
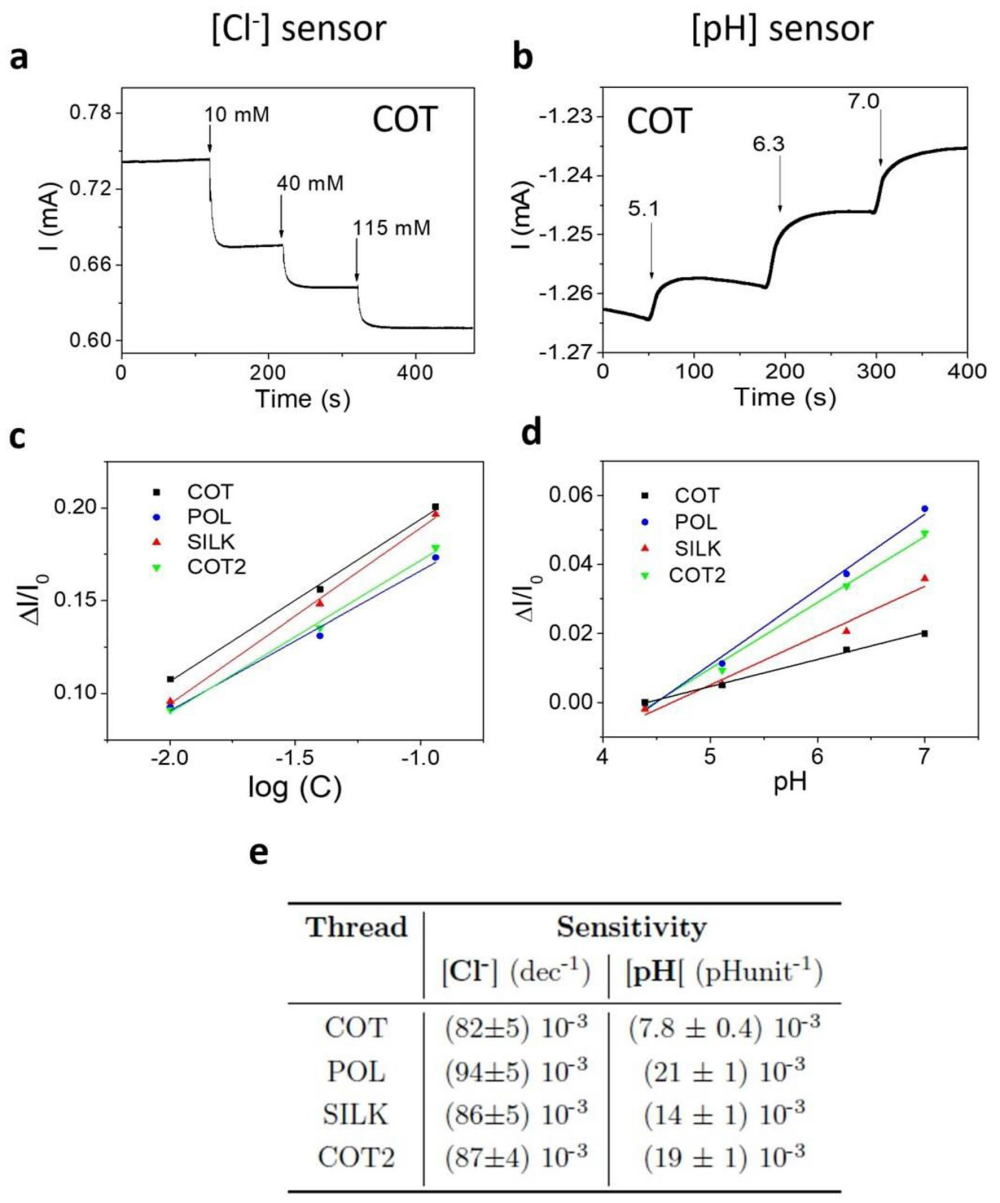
Current vs time plots recorded in 10 mL of Universal buffer: **(a)** COT Cl^−^ sensor response in the chloride relevant concentration range of human sweat between 10 and 100 mM. The Cl^−^ concentration has been increased adding 2 M of KCl in the whole solution. The applied potential is 0.1 V. (**b**) COT pH sensor response in the pH relevant range for human sweat between 5 and 7 pH level. The pH has been increased adding 1 M of KOH. The applied potential is − 0.2 V. Linear response of the normalized current variation versus the (**c**) logarithm of chloride ion concentration and (**d**) pH value, for the four yarns. Each thread was characterized separately. The error bars are omitted as the relative error is less than 1%. (**e)** Summary of threads sensitivities for all the different analysed fibers.

The two-terminal Cl^−^ sensor operates at a bias of 0.1 V and the chloride ion concentration was varied in the physiological relevant range of human sweat using 2 M KCl solution. This potential value is used to characterize the Cl^−^ sensors throughout this study. The UB presents an initial concentration of 0.1 mM Cl^−^ used as normalizing value for the current signal. Figure 3a shows the current flowing through an Ag/AgCl NPs functionalized cotton thread, as function of time, with the arrows highlighting the variation, upon increasing additions, of Cl^−^ concentration.

On the other hand, the two-terminal pH sensors were operated at a bias of − 0.2 V and the current value recorded at the initial pH 4.2 was used to normalize the signal. This potential value is used to characterize the pH sensors throughout this study. The pH level was studied in the physiologically relevant range from 4 to 7, adding 1 M KOH to the buffered solution. Figure 3b shows the current flowing through a PEDOT:BTB functionalized cotton thread, as function of time, with the arrows underlining the variation, upon KOH additions, of pH level. The observed sensitivities vary with the fiber substrate and are likely affected by the chemical nature and surface treatment of the commercial yarns.

It is worthy to note that we have not observed any kind of chemical contamination between the functionalized textile-thread sensors and the overall solution that can affect the sensing of the target analytes. In order to avoid any possible contamination of the sample solution, the sensors were always thoroughly rinsed with doubly distilled water after the electrodeposition step.

For the BTB-functionalized thread sensor, we did not observe any coloration of the solution under investigation after several measuring hours, thus confirming that the release of BTB dye is negligible, if even present. In addition, the Ag/AgCl is a well-known and wide spread material approved for the use on skin in various medical application, such as ECG^55^ and EEG^56^ electrodes, and it can be considered non-toxic^57^ in the framework of the final application of these sensors. Furthermore, according to the solubility equilibrium of silver chloride in water-based solution, the eventually released of nanoparticles would not influence the Cl^−^ sensor signal. Figure S2, in the Supplementary Information, presents the response of all the different fibers. Excellent sensing performances were found for both sensors types and Fig. 3c,d show the linear relationship between the normalised current variation and log [Cl^−^] or pH, respectively. The table reported in Fig. 3e shows the sensitivities found for the different sensors and yarns. In detail, it highlights the accuracy and the comparable behaviour of the sensors despite the threads types. Moreover, POL and COT based sensors show the highest and lowest sensitivities, respectively, for both chloride and pH detection. The average response time (assessed as the time required to reach the 90% of the final signal) is equal to (19 ± 6) s for a 115 mM chloride addition and (190 ± 30) s for a KHO addition to reach the 7.1 pH level. These values are in agreement with the previous reported results^22,58^. As regards the two types of cotton threads, despite the sensitivity to chloride is comparable for both COT and COT2, COT shows a considerably lower sensitivity to pH than COT2.

Even though a complete investigation was beyond the purposes of this work and will be the subject of further studies, we hypothesized that the reason may be found in the different ways PEDOT:PSS is adsorbed by the threads and fills inter-fibers spaces as showed on Supplementary Fig. S3. The COT thread presents higher conductivity (**G**) than COT2 but exhibits, for pH sensors, a lower sensitivity (S). We suggest that this behaviour is related to the different linear density of the two threads. The Numeric Count, which is a standard parameter to express the size of a cotton yarn, was ρ_L_ = 20 g/km and ρ_L_ = 33 g/km, for COT and COT2, respectively. Assuming the same section for every fiber and the same volumetric density for both yarns (made of the same material), COT presents fewer fibers than COT2. The schematic and simplified representation is reported in Fig. S3a. For this reason, a higher quantity of conducting polymer PEDOT:PSS coats the COT thread, still without wetting the “bulk” of the thread. Accordingly, a lower resistance is reported for COT than COT2.

On the other hand, Ag/AgCl Nanoparticles (Fig. S3b) and PEDOT:BTB (Fig. S3c) differently functionalize the PEDOT:PSS coated threads. Ag/AgCl NPs, owing to the small diameter, succeed in functionalizing almost all the PEDOT:PSS, whereas the PEDOT:BTB acts as a surface coverage^54^, leaving the polymeric material between the fibres in its pristine state (see the inset in Fig. S3c). Taking into account the Universal Buffer, Cl^−^ sensors report closer sensitivities for the two yarns, while pH sensors based on COT show halved sensitivities than COT2, possibly due to the unfunctionalized PEDOT:PSS present in the spaces among the outer separated fibres.

Repeatability and reproducibility of the different threads, for chloride and pH detection, were assessed showing promising results.

**Figure 4:**
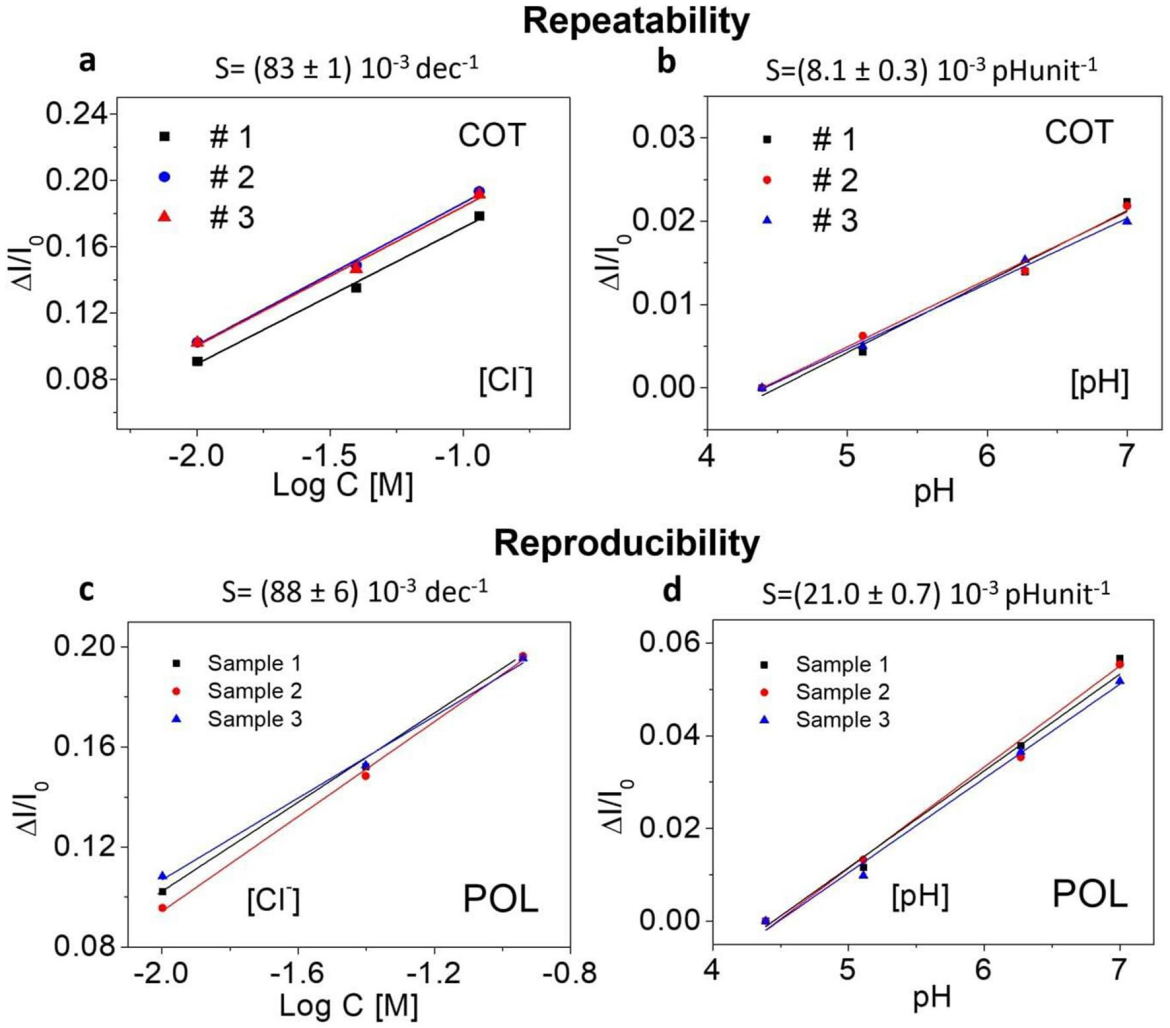
Repeatability and reproducibility of yarns: repeatability of COT fiber using the same (**a**) Ag/AgCl NPs and (**b**) PEDOT:BTB-functionalized device tested three consecutive times. The sensitivity value extracted from the linear fit is reported on the top of the graph. Reproducibility of three different samples, realized using the same type of fiber (POL), functionalized with (**c**) Ag/AgCl NPs and (**d**) PEDOT:BTB. All measurements have been carried out in 10 mL of Universal buffer.

As an example, Fig. 4a,b show repeatability studies carried out using a cotton thread, for chloride and pH measurement. To this aim, the same thread was successfully used to make three successive and consecutive experiments, giving almost superimposable response.

As far as the reproducibility, Fig. 4c,d show the results of the experiments conducted over three different POL thread for Cl^−^ and pH detection. Moreover, the reproducibility is very high as pointed out by the low standard deviation associated to the slopes of the calibration plots.

**Figure 5:**
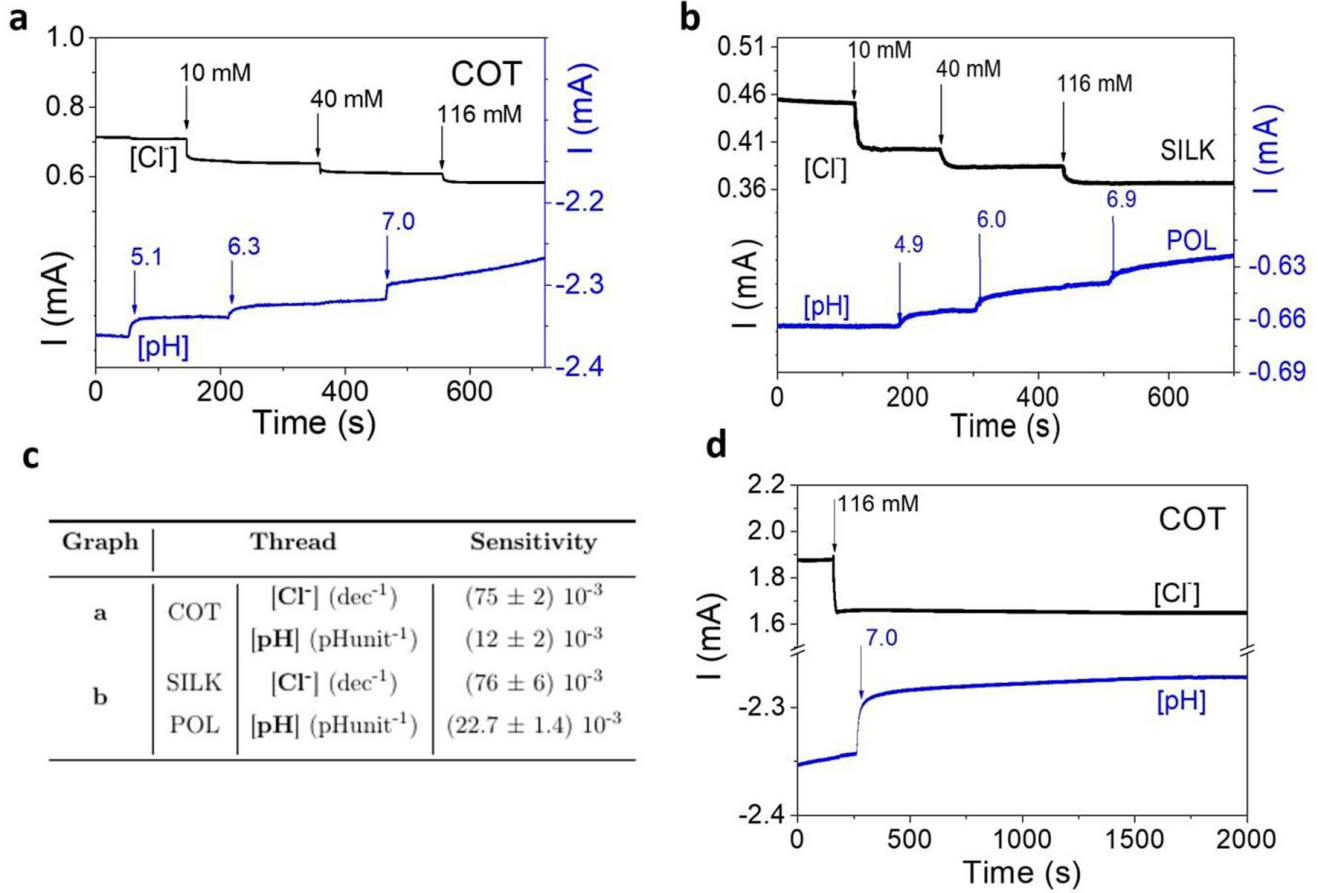
Assessment of possible interference effects for two threads immersed in the same volume of 10 mL of Universal buffer: (**a)** Response after subsequent additions of 1 M KOH and 2 M KCl for two single COT thread sensors functionalized to detect Cl^−^ and pH. Both chloride concentration and pH value range are chosen in the psionically relevant range of human sweat (Cl^−^: 10 mM to 115 mM; pH from 5 to 7). (**b)** Response after subsequent additions of 2 M KCl and 1 M KOH for silk and polyester thread sensors functionalized to detect Cl^−^ and pH sensor, respectively. The Cl^−^ sensors are reported in black, while the pH ones in blue. In the two measurements, the order of the additions was exchanged to properly asses the lack of interference effects. (**c)** Extracted sensitivities of the characterized sensors. (**d)** Long-term stability of COT thread sensors for Cl^−^ (black line) and pH level (blue line) acquired in 10 mL of Universal buffer to evaluate the stability of the signal.

Since the target of this work is the development of next-gen textile sensors, we assessed the ability of the here reported two-terminal Cl^−^ and pH sensors to work in parallel for simultaneous and selective data collection from the same analysed solution. Their selectivity, chemical interference and cross-talk effects are crucial features for wearable sensors that have to deal with simultaneous variation of different analytes and metabolites in the same medium. Since sweat is a complex mixture of several compounds, the ability to discriminate different stimuli is a fundamental property to define the performance of a sensor. To investigate the potential interference of our multi-textile thread platform, the current responses of two single thread two-terminal sensors were measured simultaneously (Fig. 5a,b). Both 1 M KOH and 2 M KCl were alternately added to 10 mL of UB. Figure 5a shows the response of two sensors realized with the same fiber (COT), while Fig. 5b proves the effectiveness of employing sensors based on different fibers together (i.e. SILK and POL for the Cl^−^ concentration and pH monitoring, respectively).

As shown in the Supplementary Fig. S4 and reported in Fig. 5, when the single thread sensors work in parallel and in the same Universal Buffer solution, they perform the same way in terms of stability, selectivity and sensitivity, compared to when they are characterized separately with no cross-talking effects and interference.

Therefore, multiplexed and quantitative detection of the two analytes can be carried out simultaneously and with no need to perform output signal processing or correction. These results assess the possibility to integrate the sensors directly into a fabric, choosing the fiber substrate according to the desired specifications in terms of sensitivity and material compatibility. It is worth noting that both natural and synthetic fibers can be exploited to realize a complete textile platform to monitor chloride ion concentration and pH level after a proper functionalization procedure.

Both types of sensors, fabricated on COT yarns, were further tested to examine the long-term stability by evaluating the eventual current drift. As reported in Fig. 5d, the sensors show a very low drift in the signal, pointing out the good long-term monitoring capability of the Cl^−^ and pH sensors.

### Artificial sweat characterization

**Figure 6:**
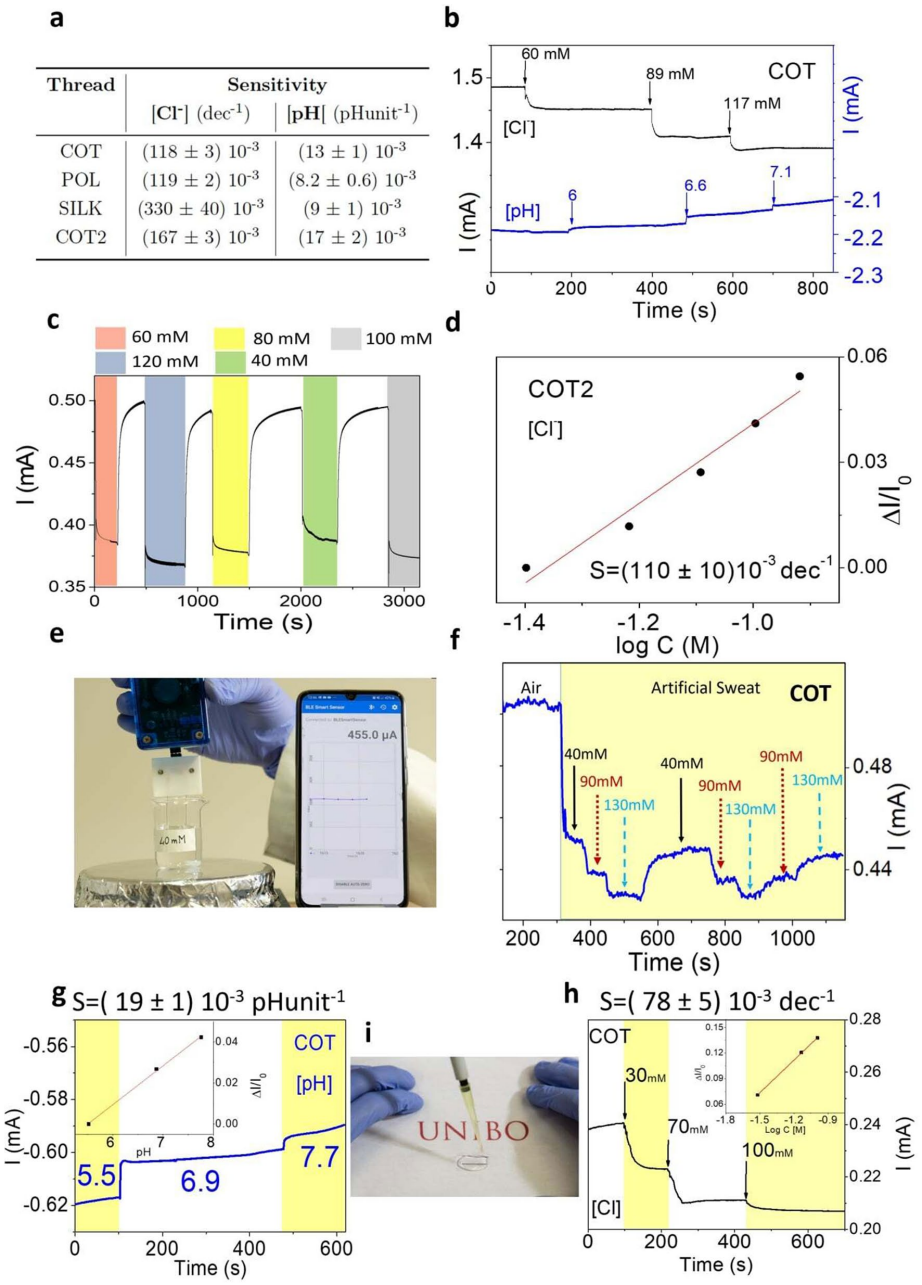
Characterization in artificial sweat and applications: **(a**) Table reporting the sensitivities of the single thread characterization in 10 mL of Artificial Sweat. For the Cl^−^ and pH thread sensors the current is normalised with the I_0_ value acquired at 40 mM and 5.5 pH level, respectively. (**b**) Interference test in 10 mL of Artificial Sweat to simulate real-life condition using COT threads. The sensitivities values in artificial sweat are (141 ± 8) 10^–3^ dec^−1^ and (18 ± 2) 10^–3^ pH unit^−1^ for Cl^−^ and pH sensors, respectively. (**c**) Random immersion of the COT Cl^−^ sensor in chloride ion solutions having different concentrations. The white parts indicate the thread sensor in air, while the coloured parts point out the thread immersed in liquid. (**d)** Calibration plot of the COT thread as Cl^−^ sensor after random additions while using the wireless-portable read-out electronics. (**e)** Test of Cl^−^ concentration in artificial sweat with a custom wireless data-reader. **(f)** Plot of the signal shown on a smartphone after consecutive addition of 3 M KCl (40 mM, 90 mM, 130 mM). The arrows indicate the interval in which there is a variation in the Cl^−^ concentration. The reversibility test has been carried out by bringing the Cl^−^ concentration back to the starting value. Current signal, calibration plot and sensitivity for (**g)** pH sensor and (**h)** Cl^−^ sensor. The threads are immersed in 1 mL of artificial sweat an 1 M KOH and 3 M KCl were used to change the pH level and Cl concentration, respectively. (**i**) Example of a sensorized T-shirt for hydration and pH monitoring used to characterize the textile thread sensors in conditions similar to the real ones.

A further relevant demonstration of the reliability of the here reported Cl^−^ and pH textile sensors was achieved by assessing their performance in artificial sweat solution, which excellently simulates the real-life sweat composition (ISO pH 5.5). Figure 6a shows the results of the single thread sensors characterization in 10 mL of artificial sweat in response to Cl^−^ concentration and pH variation. Figure S5, in Supplementary Information, summarizes the sensor electrical responses. The current acquired at 40 mM [Cl^−^] is used as I_0_ to normalize the extracted values. To be consistent with the previous cases, the performance of sensors based on COT were widely tested. Figure 6b shows the results of the interference test using two different sensors for simultaneous detection of Cl^−^ and pH in the same solution. In this case, the sensitivities of the Cl^−^ and pH sensors are only slightly higher than the one obtained in the single thread characterization, since KOH or KCl additions barely affect the Cl^−^ and pH sensors behaviour, respectively. Indeed, their values in artificial sweat are (141 ± 8) 10^–3^ dec^−1^ and (18 ± 2) 10^–3^ pH unit^−1^ for Cl^−^ and pH sensors, respectively.

Moreover, Supplementary Figure S6 reports the calibration curves of different threads sensors obtained from two separate interference tests. In both situations, no interference was observed and the sensitivities for Cl^−^ and pH sensors are in perfect agreement, highlighting the device reproducibility.

Noteworthy, the multi-sensor textile platform composed by the two functionalized threads maintained high selectivity upon varying not only the Cl^−^ concentration or pH value, but also the concentration of compounds that are usually present in human perspiration. Figure S7 (see Supplementary Information) reports the addition of urea, ethanol and lactate at their typical concentration in human sweat^41,59–61^. In all the studied cases, no interference occurred in the determination of Cl^−^ concentration.

To ensure the reliability of the sensors, we randomly dipped the Cl^−^ thread sensor in 10 mL of artificial sweat with different Cl^−^ content to extract a calibration curve, which was comparable to the results obtained with the standard characterization. The sensor response is shown in Fig. 6c while the its calibration plot and sensitivity are reported in Fig. 6d. The current dramatically increases when the thread is removed from the solution and left in air, returning to its initial value. After each immersion, the current value decreases and rapidly stabilizes accordingly to the specific chloride ion concentration. This test allows us to assert that hysteresis and memory-like effects can be neglected in our sensors. The feasibility and wearability of the Cl^−^ thread sensor was demonstrated using a portable wireless data-reader connected via Bluetooth with a smartphone. The custom application, shown in Fig. 6e, allows monitoring and recording in real-time the current signal. Figure 6f reports the characterization plot of the single cotton thread showing the reversibility of the response after variation of Cl^−^ concentration mimicking a real experiment in liquid environment. In addition, different frames of a continuous Cl^−^ concentration monitoring are reported in the Supplementary Fig. S8 in which the recovery ability and reliability of the sensor is proved.

Herein, the two types of sensing threads have been sewn into the neck of a T-shirt (Fig. 6h) to perform an in-situ sweat analysis of Cl^−^ concentration and pH level. We provide the proof of concept and an applicative example that the threads can be sewn in a fabric and continuously monitor in real-time analytes concentration. Since we demonstrated that the sensors response does not depend on volume, our sensors platform is also able to work with very low amount of human perspiration. In this case, we soak the COT threads with 1 mL of artificial sweat and we add independently 1 M KOH to change the pH from 5.5 to 7.7 and 3 M KCl to cover the chloride concentration from 30 to 100 mM. In this first stage, using a hydrophobic T-shirt allow us to work without a microfluidics system that will be required for further developments. Figure 6g–i report the acquired signal, the calibration plot using normalised current and the sensitivities values of the pH and Cl^−^ sensors, respectively. Even in these conditions, more similar to the real one, the sensors show good performance comparable to the one obtained in laboratory environment with a sensitivity for the Cl^−^ and pH sensor of (19 ± 1) 10^–3^ pH unit^−1^ and (78 ± 5) 10^–3^ dec^−1^, respectively.

Finally, the here reported study shows the possibility to realize textile multi-sensor devices able to simultaneously measure the pH and Cl^−^ concentration in the same solution without interferences or the need of shielding to avoid sample cross-talk.

In the last years, several electrochemical textile devices, able to monitor only the pH of sweat in a linear range physiologically relevant for human biofluids, have been proposed (see Table 1). Similarly to other cited studies, our two-terminal sensors keep the robustness of the potentiometric-like transduction as explained in our previous works^37,54^.

**Table 1:** Main features of some textile and wearable sensors for pH level and Chloride ions.

	Active material	Textile support	Transduction mechanism	Sensitivity	Characterization solution	Linear range	Ref.
pH	PEDOT:BTB	Cotton, silk and polyester thread	Electrochemically gated	(13 ± 1) 10^–3^ pH unit^−1^ for Cotton	Buffer/artificial sweat	4–7	This work
	Iridium oxide film	Nylon-based conducting fabric	Potentiometric	47.54 mV/pH	Buffer/real sweat	4–8	^65^
	Thick film graphite-polyurethane composite	cellulose-polyester blend cloth	Potentiometric	4 mV/pH	Buffer	6–9	^66^
	PANi	elastomeric gold fibers woven into a textile matrix	Potentiometric	60.6 mV/pH	Buffer/artificial sweat	4–8	^67^
Cl	Ag/AgCl NPs	Cotton, silk and polyester thread	Electrochemically gated	(167 ± 3) 10^–3^ dec^−1^ for Cotton	Buffer/artificial sweat	10–115 mM	This work
	Ag ink	PDMS	Potentiometric	− 51.02 mV/dec^−1^	NaCl, artificial sweat	10–160 mM	^62^
	PEDOT:PSS	–	Organic electrochemical transistor	150 10^–3^ dec^−1^	PBS, artificial and human sweat	1 mM–1 M	^63^
	Ag NPs	PET/Gore-Tex	Voltammetric	292.2 µA M^−1^	Synthetic sweat	5–60 mM	^64^

To the best of our knowledge, the here reported Cl^−^ thread sensors is the first example of a selective and fully-textile thread device that does not need a reference electrode and is able to simultaneously operate 2 sensors immersed in the same solution, without interferences . We reported in Table 1 the performance of up-to-date wearable chemical sensors. In particular, in 2019 Xu et al.^62^, reported a electrochemically wearable Cl^−^ sensors that could be used in parallel with a Ca^2+^ sensor patch showing a sub-nerstian sensitivity and a limit of detection slightly lower than ours (see Fig. S9 in Supplementary).

Kim et al.^63^ described a two-terminal PEDOT:PSS thread that can be sewn into a fabric and non-selectively sense total cations concentration. Bujes-Garrido et al.^64^ reported voltammetric screen-printed three-terminal Cl^−^ sensors with AgNPs deposited onto the working electrode, employing both PET and Gore-Tex as substrate. Despite the low limit of detection of 5 µM, it exhibits a narrow operation range 5–60 mM, thus limiting its operation to applications where small concentration variations are expected.

## Conclusion

The development of textile multi-sensor arrays is an advanced technological frontier in the design of tools for non-invasive monitoring of physiological parameters in humans. By exploiting a new concept of electrochemical transduction, we have here reported a novel example of multi-analyte textile platform for the continuous detection of pH and chloride in human perspiration. Each sensor is a two-terminal device composed by a single thread, working in aqueous solution without the need of a reference or gate electrode, with a significant simplification of the final device with respect to amperometric, potentiometric or transistor configuration. The sensitive yarns are realized using commercial textile fibres, both natural and synthetic, coated with a semiconducting polymer, properly functionalized to selectively detect chloride ion concentration and pH level in sweat. The reliability of the sensors was demonstrated first in universal buffer and then in artificial sweat, in order to mimic a real-life application environment. The sensors show stability, reproducibility and repeatability for all of the studied fibers and there are not significant difference between the fibres in terms of the overall performance, drawing the attention on the possibility to use the proper threads according to final product. Their ability to work in parallel, without interfering or change sensitivity, paves the ways for simultaneous and real-time Cl^−^ concentration and pH level monitoring in body fluid, such as sweat.

This work provides the technological basis for the fabrication of single-thread sensitive elements and describes their assembly in a multi-platform biosensor system. Nevertheless, it still needs further improvement for the use in real-life applications. On one hand, a reliable sampling system should be employed to regenerate sweat on sensors surface and to ensure a very continuous monitoring. On the other hand, new functionalization should be designed to widen the bio-compounds that can be detected with this technological approach.

## Supplementary information

Supplementary Information.
