# Supplementary material for: Textile sensors platform for the selective and simultaneous detection of chloride ion and pH in sweat
Source: Sci Rep. 2020 Oct 14;10:17180. doi: 10.1038/s41598-020-74337-w (PMC7560666; doi:10.1038/s41598-020-74337-w)
Supplement: Supplementary file 1 — Supplementary Information. [file 41598_2020_74337_MOESM1_ESM.docx]

**Supporting Information**

**Textile sensors platform for the selective and simultaneous detection of chloride ion and pH in sweat**

Luca Possanzini^1^, Francesco Decataldo^1^, Federica Mariani^2^, Isacco Gualandi^2^, Marta Tessarolo^1^, Erika Scavetta^2^ and Beatrice Fraboni^1^

^1^ Department of Physics and Astronomy, University of Bologna, Viale Berti Pichat 6/2, 40127, Bologna, Italy.

^2^ Department of Industrial Chemistry, University of Bologna, Viale Risorgimento 4, 40136, Bologna, Italy.

*Corresponding author: luca.possanzini2@unibo.it


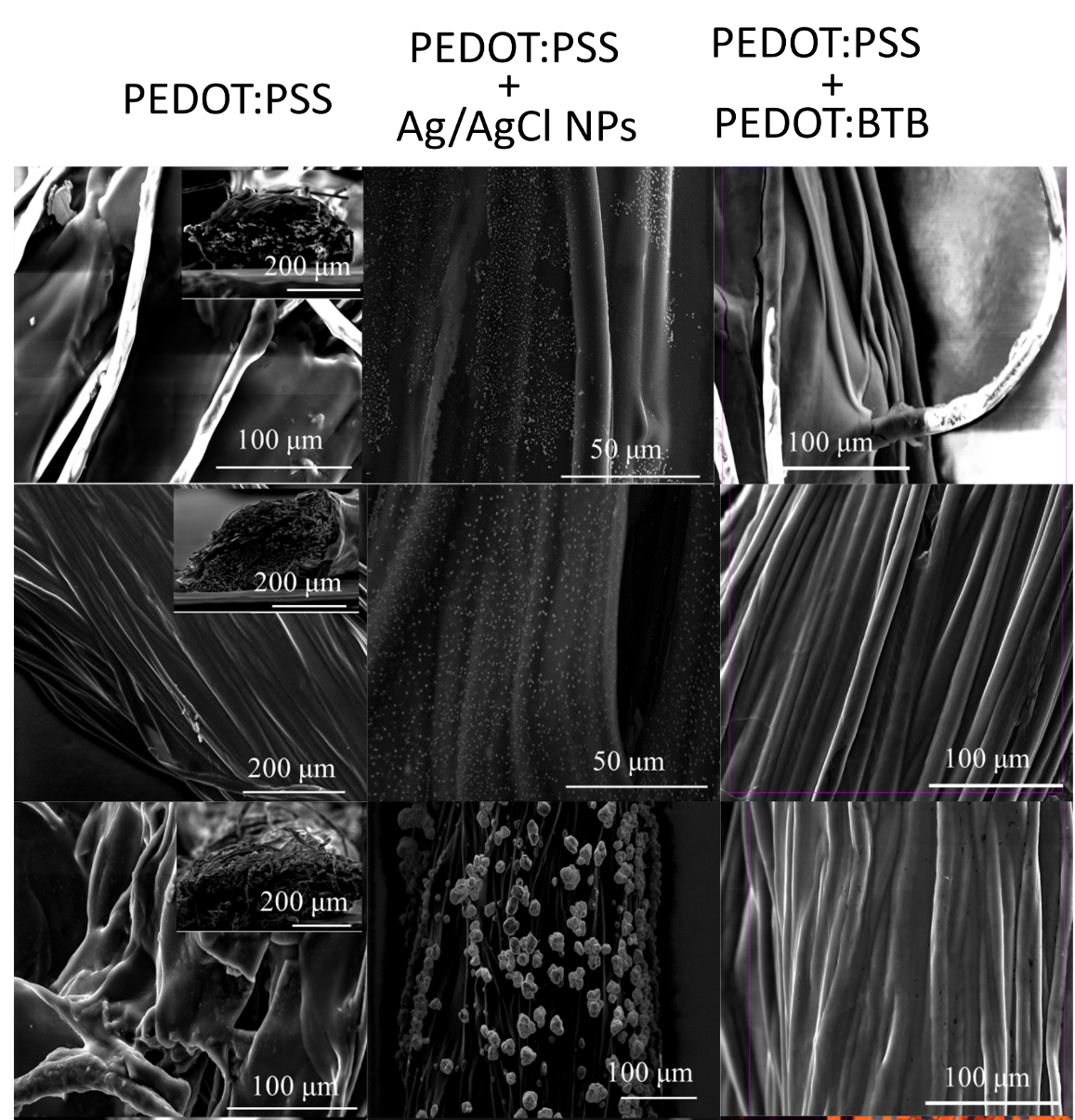


**Figure S1** SEM images of the sensorized threads. Thread coated with PEDOT:PSS (left column), with electrodeposited Ag/AgCl NPs (middle column) and with PEDOT:BTB (right column). Each row corresponds to a different kind of yarn: Polyester (first), Silk (second) and cotton (third).

POL

SILK

COT

**
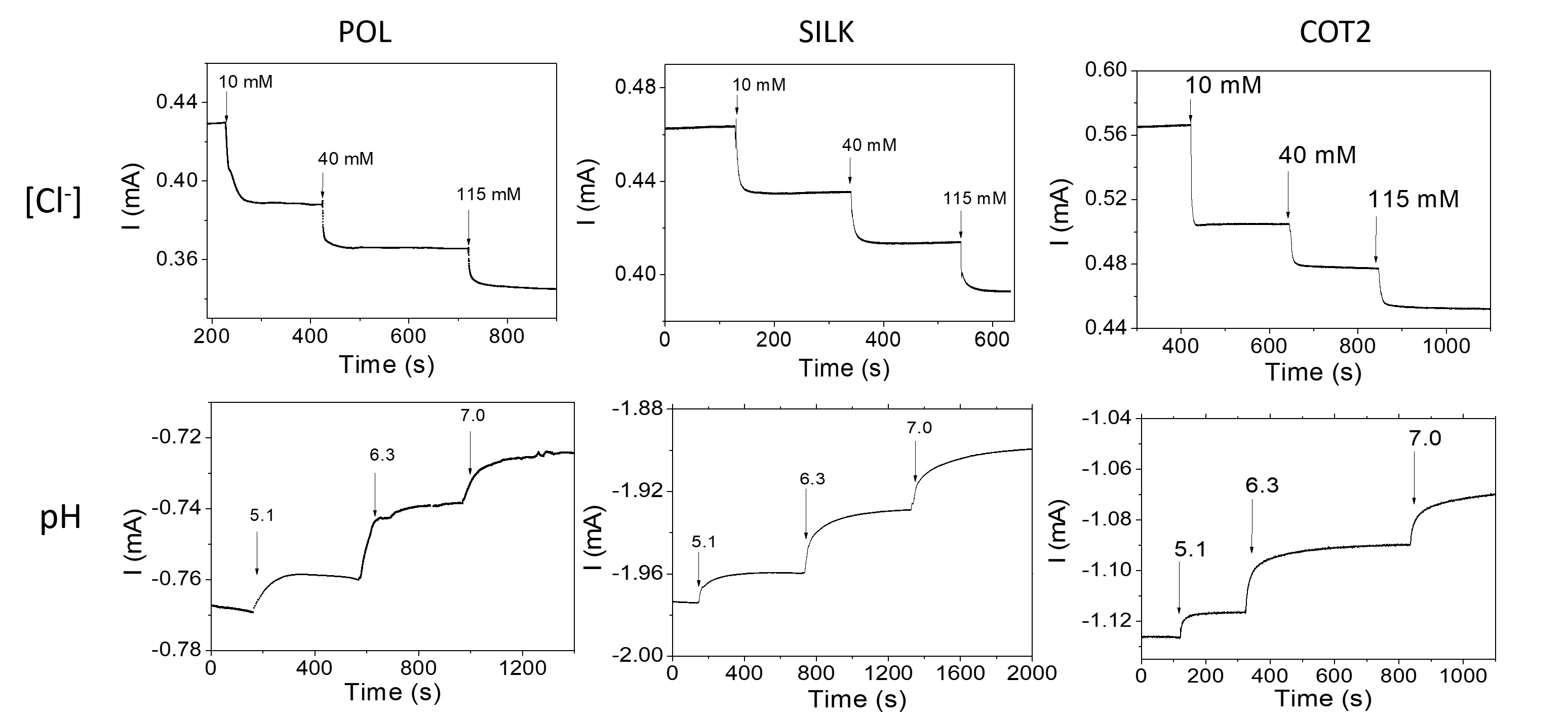
**

**Figure S2** Electrical response of the different thread sensors in 10 mL of Universal Buffer. The first row reports the [Cl^-^] thread sensors after the increments of Cl concentration, while the second row reports the response of pH thread sensors after the addition ok KOH which change the pH value.


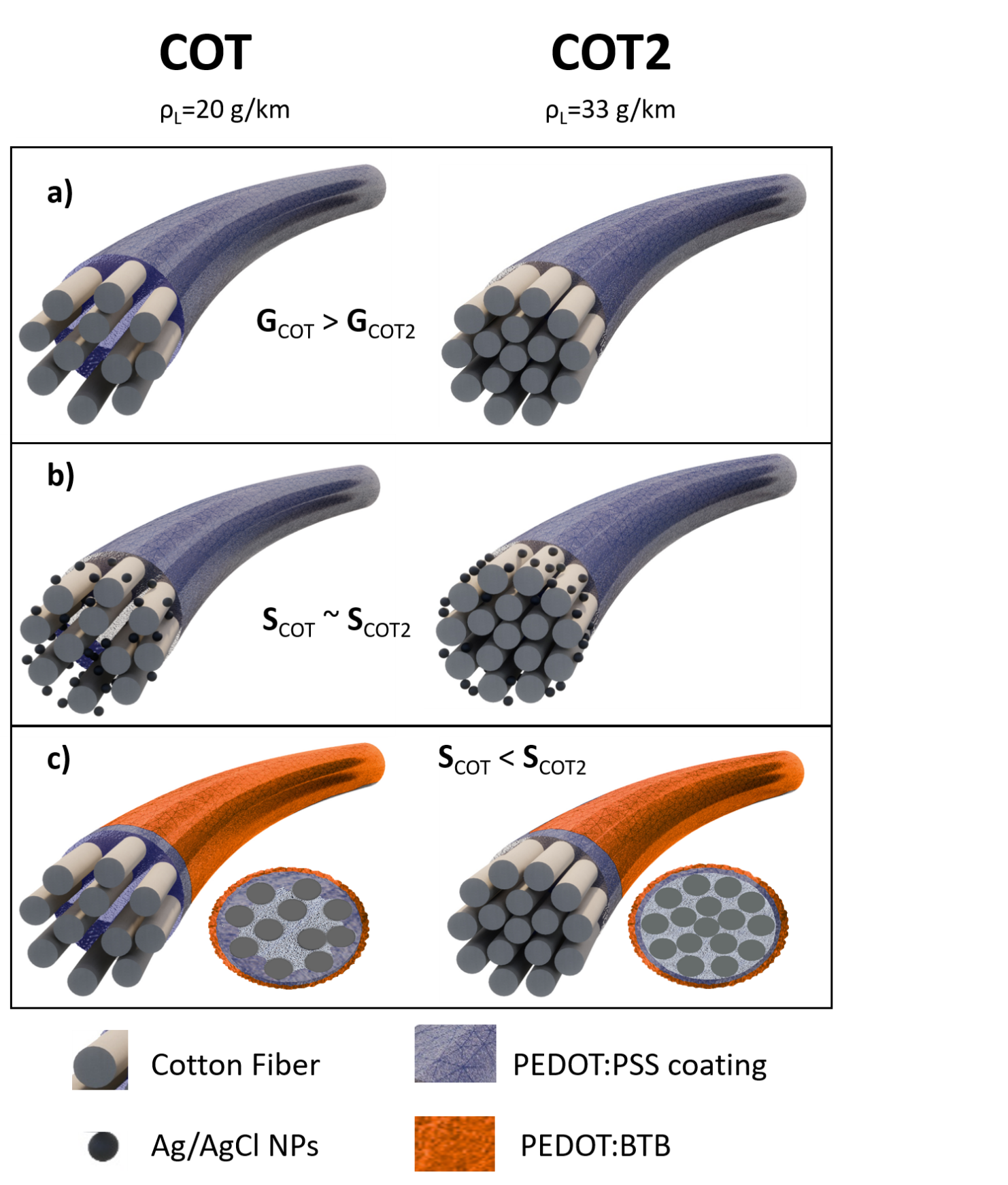


**Figure S3:** Schematic view of the proposed COT and COT2 polymeric coating (a), Ag/AgCl nanoparticles (b), and PEDOT:BTB functionalization (c), with the consequent relative comparison of conductivity and sensitivity. **G** and **S** represent the conductivity and the sensitivity, respectively.

**Figure S4** Normalized current versus the logarithm of chloride ion concentration and pH unit for two different interference tests in 10 mL of Universal Buffer.


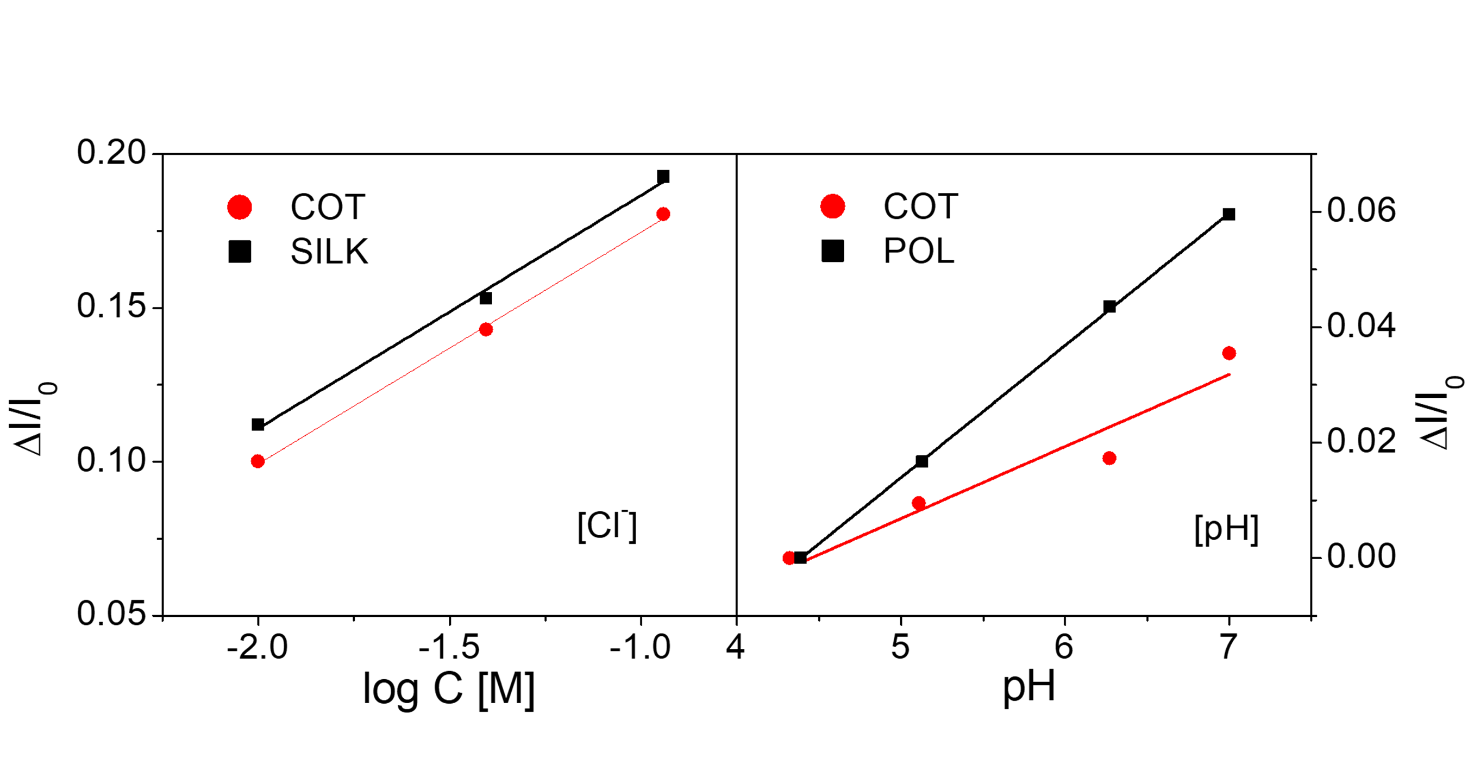

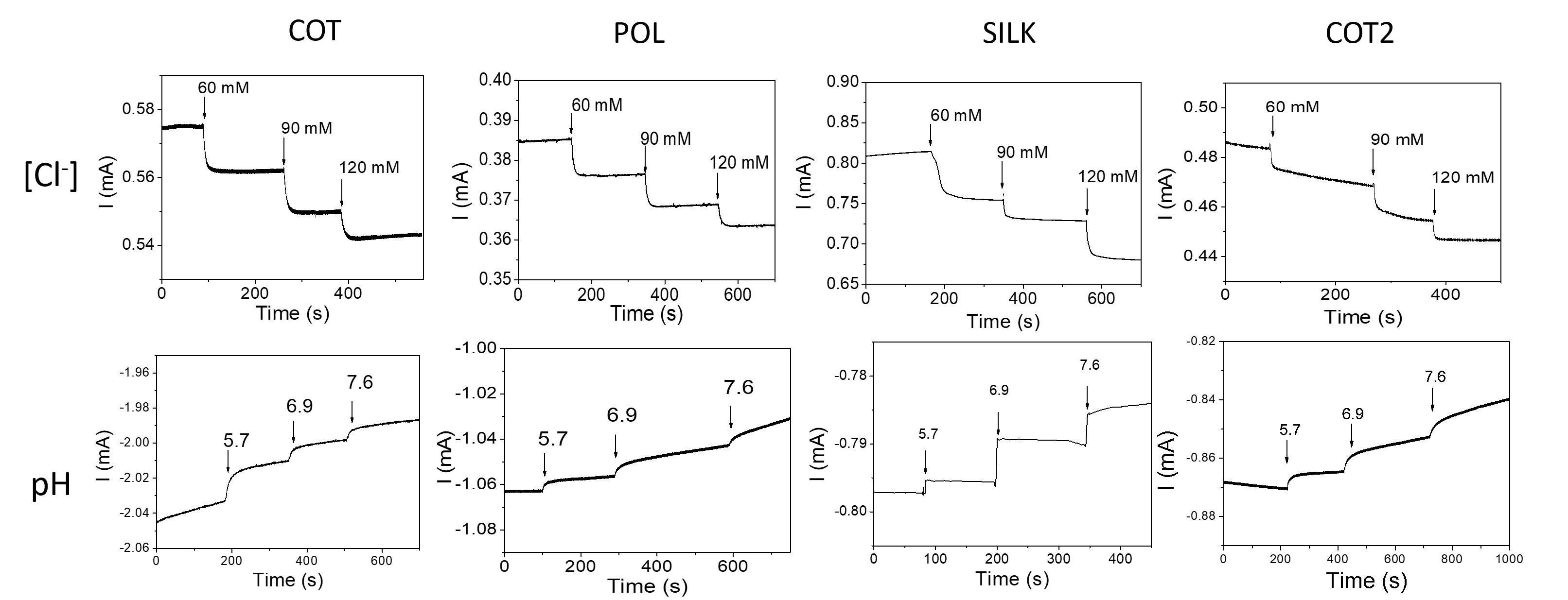


**Figure S5** Electrical response of the different thread sensors in 10 mL of Artificial Sweat. The first row reports the Cl^-^ thread sensors response after the increments of chloride concentration, while the second row reports the response of pH thread sensors after the addition of KOH which change the pH value.


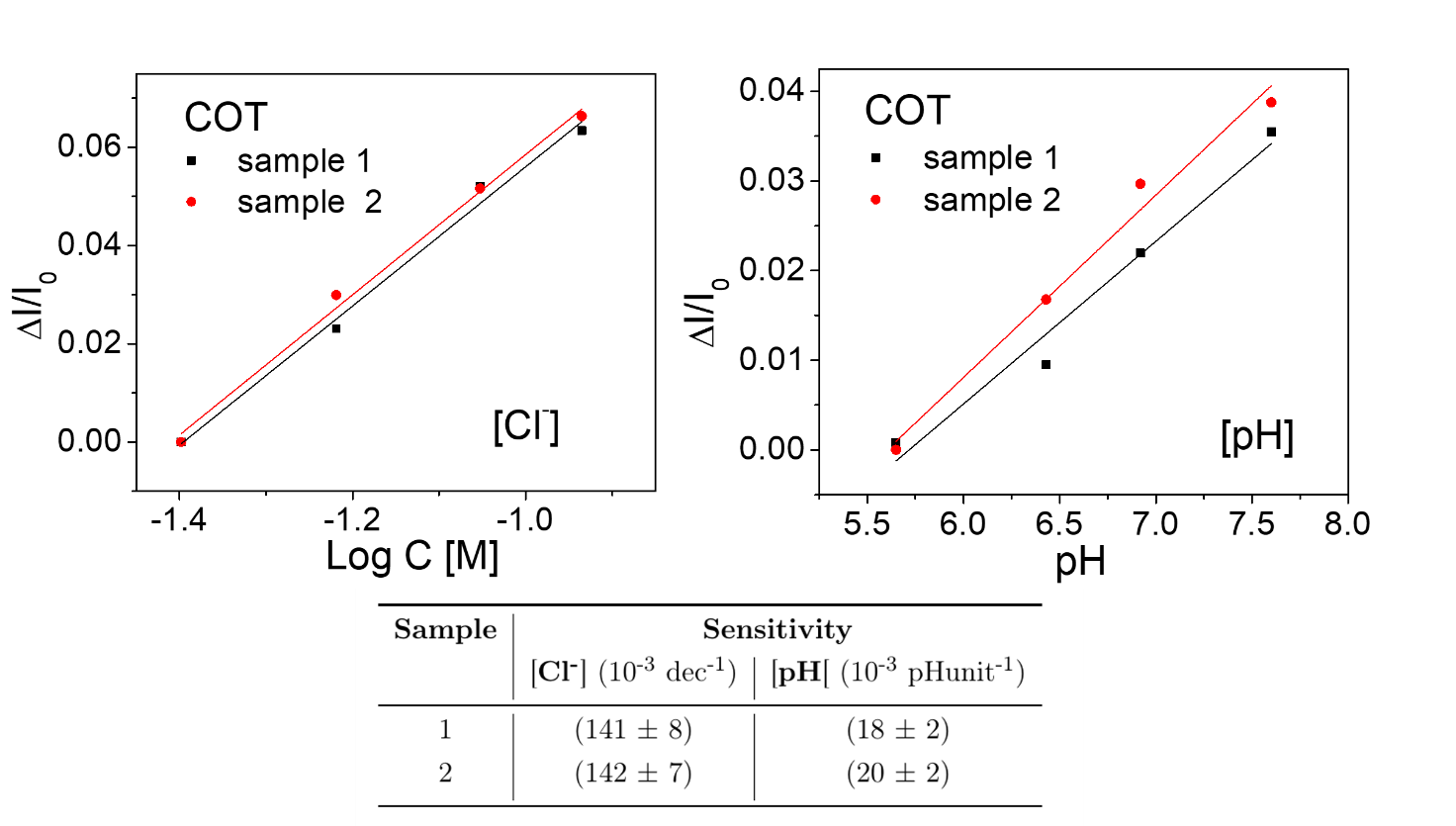


**Figure S6** Calibration curves of different sensors based on cotton threads obtained from two separate interference tests to show the reproducibility behaviour also during interference test.


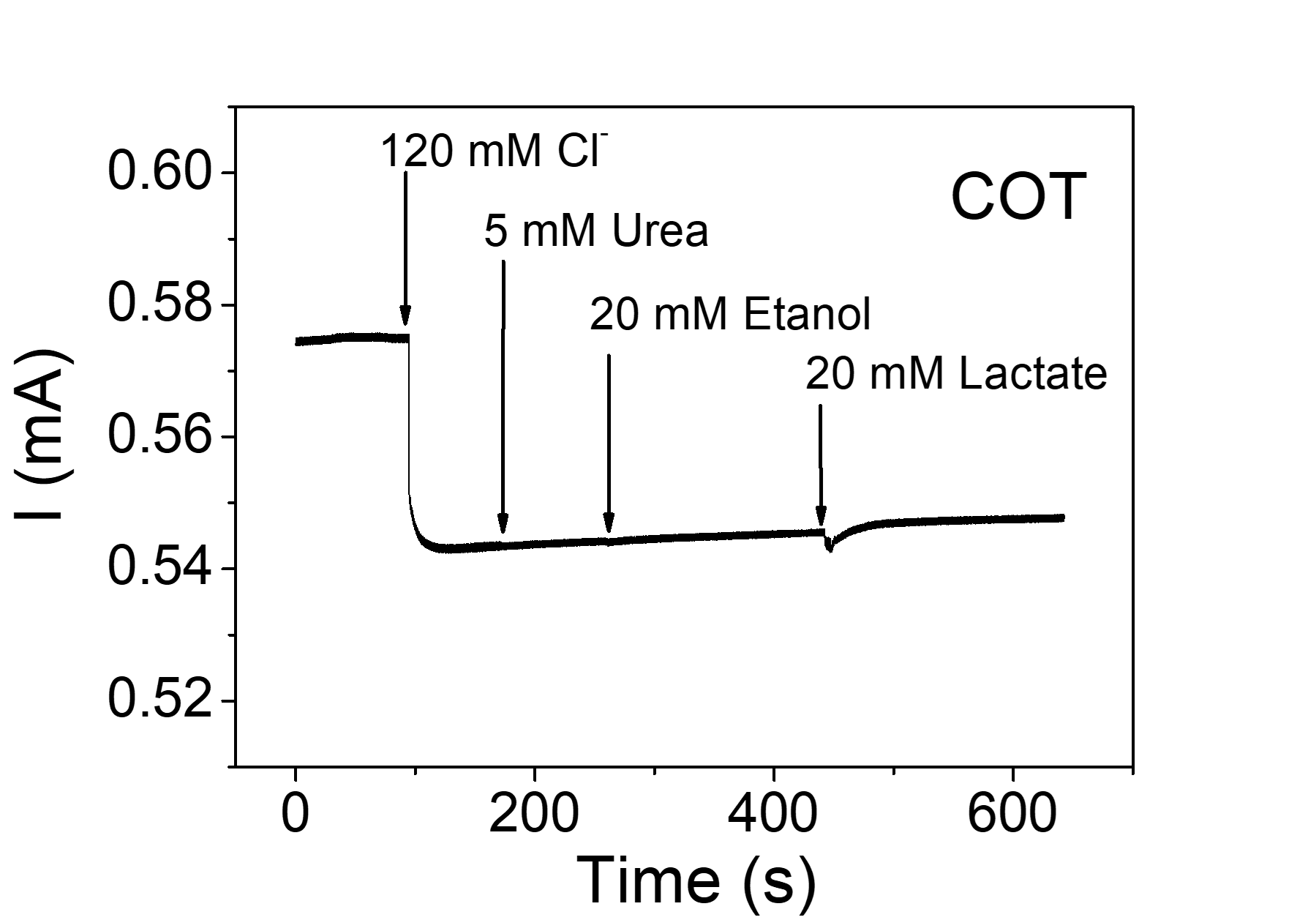


**Figure S7** Selectivity test to investigate the possible interference of different compounds evaluated at their typical concentration in artificial sweat.


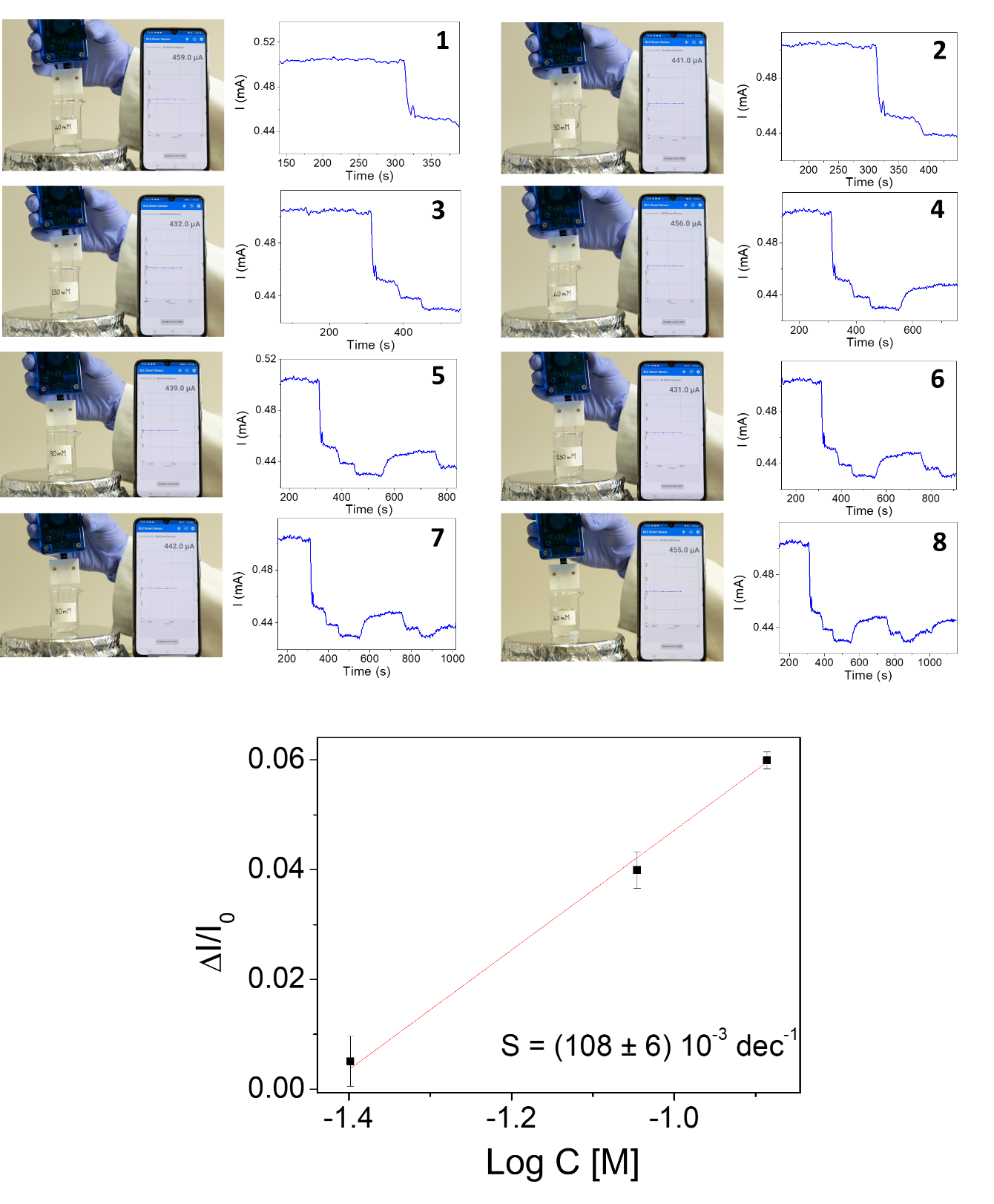


**Figure S8** Consecutive snapped frames during a real-time simulation test of the chloride ions concentration cotton textile-thread sensor. The current signal is recorded with a portable wireless device connect via Bluetooth with a smartphone. We randomly dipped the sensor in three solutions with different KCl concentrations: the calibration curve is in perfect agreement with the previous results.

**Figure S9** Calculation of the limit of detection pf a Cl^-^ sensor based on COT thread. The sensors are dipped in 10 mL of 0.1 M KNO_3_ without chloride. The blank current is I_0_ + Δσ_I0_ = (356.9 ± 0.2) µA. 2M KCl solution is used to increase the Cl^-^ concentration.

The calculated value is 0.3mM and it is enough to sense the Cl- content in human sweat witch is generally comprise between 10mM to 120mM.
